# Effectiveness of trauma team on medical resource utilization and quality of care for patients with major trauma

**DOI:** 10.1186/s12913-017-2429-3

**Published:** 2017-07-24

**Authors:** Chih-Jung Wang, Shu-Ting Yen, Shih-Fang Huang, Su-Chen Hsu, Jeremy C. Ying, Yan-Shen Shan

**Affiliations:** 1 Division of Trauma, Department of Surgery, National Cheng Kung University Hospital, No.138, Sheng Li Road, Tainan, 704 Taiwan, Republic of China; 2 Department of Healthcare Administration, College of Medicine, I-Shou University, No.8, Yida Road, Yanchao District, Kaohsiung, 824 Taiwan, Republic of China; 3 School of Public Health and Management, Wenzhou Medical University, Wenzhou, China

**Keywords:** Major trauma, Medical resource utilization, Quality of care

## Abstract

**Background:**

Trauma is one of the leading causes of death in Taiwan, and its medical expenditure escalated drastically. This study aimed to explore the effectiveness of trauma team, which was established in September 2010, on medical resource utilization and quality of care among major trauma patients.

**Methods:**

This was a retrospective study, using trauma registry data bank and inpatient medical service charge databases. Study subjects were major trauma patients admitted to a medical center in Tainan during 2009 and 2013, and was divided into case group (from January, 2011 to August, 2013) and comparison group (from January, 2009 to August, 2010).

**Results:**

Significant reductions in several items of medical resource utilization were identified after the establishment of trauma team. In the sub-group of patients who survived to discharge, examination, radiology and operation charges declined significantly. The radiation and examination charges reduced significantly in the subcategories of ISS = 16 ~ 24 and ISS > 24 respectively. However, no significant effectiveness on quality of care was identified.

**Conclusions:**

The establishment of trauma team is effective in containing medical resource utilization. In order to verify the effectiveness on quality of care, extended time frame and extra study subjects are needed.

## Background

Trauma is one of the major causes of death worldwide, and it is an unavoidable and constant significant issue of medical care [1]. In Taiwan, trauma due to accident ranked the top sixth cause of death. Moreover, it ranked the first and second cause of death among adolescents and age group of 15 to 44 [2].

Life threatening trauma cases should be taken care of by a team of medical specialties utilizing appropriate medical resources and under timely diagnosis/treatment within so-called “the golden 1 h” [3, 4]. The management and execution of trauma team is an integration of multi-specialties, such as trauma surgeon, anesthesia, trauma ICU, neurosurgery, reconstructive surgery, radiology, blood bank, etc., and providing the preeminent medical care around the clock, 7 days a week [5–7]. The quality of emergency medical care can be improved effectively under solid training of multi-specialties trauma team [8].

Various studies had illustrated that a solid trauma care system is effective in terms of improving quality of care, reducing complications, and controlling medical resource utilization [9–11]. According to Taiwan’s National Health Insurance Agency (NHIA), over 10 million trauma cases occurred in Taiwan annually, which accounted for about one forth of ER visits; and about 300 thousand cases were hospitalized. In addition, the inpatient expenditure for trauma was over 14.1 billion NT dollars per year, and total medical expenditure for trauma was more than doubled (30 billion NT dollars) [12]. However few studies examined the effectiveness of trauma care system in Taiwan.

Trauma team of the case hospital, a national university affiliated medical center in Tainan, was established in September, 2010. The trauma team is not only responsible for handling major trauma patients at the front line but also acting as the coordinator for multiple injury patients. In addition, trauma registry system and regular continuous quality improvement (CQI) meetings were implemented. No regular meeting and multi-specialty platform for multiple injury/major trauma patients were performed prior to the establishment of trauma team.

Thus, this study aimed to investigate the effectiveness of trauma team on medical resource utilization and quality of care for major trauma patients in the case hospital. The research questions as follows:

1. Are the LOS and ICU stay of the case group significant less than that of comparison group?
2. For patients who need urgent operation, is the operation waiting time (the lag time between surgeon issues the order of operation and patient was sent to OR) of the case group significant less than that of comparison group?
3. For patients who need urgent operation, is the duration stay in emergency room (the lag time between patient admitted to ER and patient was sent to OR) of the case group significant less than that of comparison group?
4. Are medical service charges (i.e., clinical charge, ward charge, examination charge, radiology charge, procedure charge, operation charge, and total medical charge) of the case group significant lower than that of comparison group?

## Methods

This was a retrospective archive study based on medical records, trauma data bank, and National Health Insurance (NHI) reimbursement data of inpatient medical service charges for major trauma patients (Injury Severity Score, ISS > 15) who sought emergency medical care at the case hospital from January, 2009 to August, 2010 (prior to the establishment of trauma team, classified as the comparison group) and from January, 2011 to August, 2013 (4 months after the establishment of trauma team, classified as the case group).

Patients with severe head and/or spinal injury, severe burn, and corrosive injury were excluded, because most of them were cared by subspecialty divisions other than trauma team. In addition, major trauma patients who sought medical care at the case hospital during September to December, 2010 were excluded due to the SOP of trauma team was in the early try-and-error stage by then. Totally 137 and 65 patients (2:1 ratio) met the inclusion and exclusion criteria, and were classified as the case and comparison groups, respectively. This study was approved by the Institutional Review Board (IRB) of case hospital. The demographic and clinical information for the patients were de-linked prior to analysis. Thus, informed consent was not obtained from patients prior to the study.

## Results

We didn’t find significant differences between the case and comparison groups, in terms of gender, age, outcome, severity, and operation. However, the proportion of urgent operation decreased by 17% after the establishment of trauma team (Table 1).

**Table 1:** Description of Subject Population Characteristics

	Before The Establishment of Trauma Team			After The Establishment of Trauma Team				
	n	%		n	%		χ^2^	p
Gender							0.007	0.933
Male	43	66.2		93	67.9			
Female	22	33.8		44	32.1			
Age			44.06 ± 19.22			42.34 ± 19.88		
< 20	6	9.2	16 ~ 83	19	13.9	0 ~ 86	1.448	0.485
20 ~ 49	31	47.7		69	50.4			
≧50	28	43.1		49	35.8			
Outcome							0.197	0.495
Survived	63	96.9		131	95.6			
Expired	2	3.1		6	4.4			
ISS							0.224	0.636
ISS = 16 ~ 24	47	72.3		93	67.9			
ISS > 24	18	27.7		44	32.1			
Underwent Operation							0.646	0.421
Yes	43	66.2		81	59.1			
No	22	33.8		56	40.9			
Urgent Operation							3.896	0.048*
Yes	18	41.86		20	24.69			
No	25	58.14		61	75.31			

*p < 0.05

No significant between-group differences in quality of care, e.g., LOS, ICU stay, operation waiting time and duration stay in ER were found. However, the total medical charge (NT$240,386 to NT$179,019) and examination charge (from NT$17,551 to NT$11,155) decreased significantly (Table 2).

**Table 2:** Differences of Medical Quality of Care and Medical Resource Utilization (All Patients)

	Before The Establishment of Trauma Team			After The Establishment of Trauma Team				
	n	Average	SD	n	Average	SD	t	p
ICU stay	49	8.33	8.86	82	8.74	8.72	−.264	0.793
LOS	65	22.11	14.86	137	19.17	15.41	1.281	0.202
Operation waiting time	18	35.39	21.06	20	28.30	53.07	.530	0.600
Duration stay in ER	18	303.17	346.62	20	160.30	140.63	1.697	0.098
Clinical Charge	65	10,152.57	7706.77	137	8554.70	7172.98	1.444	0.150
Ward Charge	65	55,002.58	57,766.82	137	43,337.06	50,819.10	1.458	0.147
Examination Charge	64	17,551.97	24,606.29	130	11,155.54	13,646.86	1.938	0.05*
Radiology Charge	64	8786.58	8979.91	128	6177.03	9066.62	1.886	0.061
Procedure Charge	65	25,158.14	29,695.98	137	21,565.15	33,210.93	.743	0.459
Operation Charge	43	54,085.60	41,041.51	93	42,826.84	35,816.98	1.627	0.106
Total Medical Charge	65	240,386.31	243,934.49	137	179,019.39	193,284.96	1.933	0.05*

*p≦0.05

In order to scrutinize the confounding of outcome and severity, we sub-categorized subjects as survived to discharge and ISS > 24, and then compare the between-group differences. The examination (from NT$15,666 to NT$10,545), radiology (from NT$8864 to NT$5638) and operation charges (from NT$54,561 to NT$41,394) decreased significantly among subjects who survived to discharge (Table 3). Significant between-group difference was also identified in examination charge (decreased from NT$ 31,917 to NT$15,731) among extremely severe trauma patients (ISS > 24). Similar results delineated that no significant between-group differences in LOS and ICU stay. However, operation waiting time among extremely severe trauma patients (ISS > 24) reduced from 35.67 to 14.4 min (Table 4).

**Table 3:** Differences of Medical Quality of Care and Medical Resource Utilization on Patients Survived to Discharge

	Before The Establishment of Trauma Team			After The Establishment of Trauma Team				
	n	Average	SD	n	Average	SD	t	p
ICU stay	47	7.81	7.96	76	8.74	8.98	−.581	0.562
LOS	63	22.16	14.80	131	19.63	15.58	1.074	0.284
Operation waiting time	17	37.47	19.71	16	33.75	58.20	.249	0.805
Duration stay in ER	17	290.65	353.06	16	180.75	141.35	1.160	0.255
Clinical Charge	63	9976.56	7433.79	131	8627.64	7289.05	1.199	0.232
Ward Charge	63	52,786.79	54,062.83	131	43,025.78	51,620.02	1.214	0.226
Examination Charge	62	15,666.29	21,999.81	124	10,545.89	13,254.21	1.975	0.05*
Radiology Charge	62	8864.85	9071.37	124	5638.11	8396.92	2.398	0.018*
Procedure Charge	63	23,629.67	27,658.80	131	21,331.63	33,887.60	.468	0.640
Operation Charge	42	54,561.48	41,418.76	88	41,394.32	34,149.59	1.916	0.05*
Total Medical Charge	63	224,411.86	227,087.79	131	172,125.21	192,091.67	1.671	0.096

*p≦0.05

**Table 4:** Differences of Medical Quality of Care and Medical Resource Utilization on Patients with ISS > 24

	Before The Establishment of Trauma Team		After The Establishment of Trauma Team					
	n	Average	SD	n	Average	SD	t	p
ICU stay	18	10.28	10.79	41	8.93	9.43	.485	0.630
LOS	18	27.94	16.54	44	23.41	19.08	.881	0.382
Operation waiting time	12	35.67	24.65	10	14.40	11.36	2.508	0.021*
Duration stay in ER	12	362.83	403.90	10	171.30	138.97	1.426	0.149
Clinical Charge	18	13,724.33	9424.68	44	10,652.98	8236.91	1.278	0.206
Ward Charge	18	82,580.44	73,395.81	44	59,647.77	58,957.53	1.293	0.201
Examination Charge	18	31,917.39	34,401.48	43	15,731.79	16,679.96	2.483	0.016*
Radiology Charge	18	10,386.78	9162.89	43	9048.88	12,255.96	.416	0.679
Procedure Charge	18	38,972.22	33,577.02	44	29,229.09	45,855.30	.815	0.182
Operation Charge	15	63,227.73	41,422.89	31	56,152.94	44,202.76	.519	0.606
Total Medical Charge	18	376,106.78	315,210.74	44	248,175.11	242,511.89	1.725	0.090

*p < 0.05

## Discussion

Implementation of trauma team can increase efficiency of medical care delivery and lower the cost [11–13]. Our study demonstrated similar results that examination charge and total medical charge decreased after the establishment of trauma team, and operation charge decreased significantly for the subgroup of survive to discharge. In addition, our results indicated that the ratio of urgent surgery among major trauma patients had decreased after the establishment of trauma team. These phenomena might due to our trauma team members had regular meeting every week and discussing complicated cases which need intervention for haemostatic and/or critical decision for life saving. Thus the trauma surgeons were familiar with the protocols for complicated injuries and experienced in assessing patients’ clinical condition. Consequently, they will be able to make clinical decision in confidence and avoided unnecessary operation.

The on duty trauma surgeon must arrive ER within 10 min after the ER physician activated trauma team for unstable trauma patients. Other team members (i.e., CT technician, anesthetist and operation room nurse) would also receive the same message. Once the system was activated, the unstable trauma patients would be treated effectively and efficiently. Our results indicated that the operation waiting time for extremely severe patients (ISS > 24) had reduced from 35 to 14 min; and the average ER duration for patients need urgent operation had declined from 303 to 160 min. Though the differences were not statistically significant due to limited case number and low statistic power, the results revealed that critical trauma patients received prompt life-saving operation after the establishment of trauma team.

Nevertheless, our study subjects were limited, and the time frame was focused on the early stage of trauma team. Thus, further studies are needed to explore the effectiveness of trauma team in terms of quality improvement with sufficient subjects and extended time frame.

## Conclusion

In conclusion, our study verified that the establishment of trauma team was effective in containing medical resource utilization, and decrease operation waiting time for extremely severe trauma patients, but had minimal effect on LOS and ICU stay. The findings of our study can be used as an imperative evidence-based reference for the case hospital and other medical centers in evaluating trauma team and other clinical taskforces.

## Abbreviations

AIS: Abbreviated Injury Scale
COD: Cause of death
CQI: Continuous Quality Improvement
DOHW: Department of Health and Welfare
DRG: Diagnosis Related Groups
ER: Emergency room
GI: Gastrointestinal tract
ICU: Intensive care unit
IRB: the Institutional Review Board
ISS: Injury Severity Score
LOS: Length of stay
NHI: National Health Insurance
NHIA: National Health Insurance Agency
NT dollars: New Taiwan dollars
NTDB: National Trauma Data Bank
OR: Operation room
SOP: Standard operation procedure
WHO: World Health Organization
